# Advancements in Acne Scar Treatment: Exploring Novel Therapies

**DOI:** 10.1111/jocd.70183

**Published:** 2025-05-02

**Authors:** Seyedeh Hoda Qoreishi, Nasim Gholizadeh, Ghasem Rahmatpour Rokni, Mahsa Babaei

**Affiliations:** ^1^ School of Medicine Islamic Azad University Tonekabon Iran; ^2^ Department of Dermatology Mazandaran University of Medical Sciences Sari Iran

**Keywords:** acne vulgaris, scar, therapeutics

## Abstract

**Background:**

Acne‐induced scarring remains a formidable challenge, compelling patients to explore surgical interventions. This study aims to delve into and showcase recent strides in acne scar treatment, with a specific focus on innovative therapies.

**Methods:**

Conducting a narrative review spanning studies from 2013 to 2023, this paper provides a general overview of interventions. The review included English‐language studies with full‐text availability. Rigorous data screening and extraction by two independent authors covered diverse facets, including study particulars, participant profiles, measurement tools, intervention durations, outcomes, and key findings. The initial search was performed within PubMed, Scopus, Google Scholar, and the gray literature.

**Results:**

Ultimately, 26 studies meeting inclusion criteria were included and reviewed for final analysis.

**Conclusion:**

Recognizing the varied nature of acne scars, this narrative review underscores the need for personalized treatment strategies, considering scar type and severity. The study also emphasizes the potential necessity of combining treatments for optimal results. Despite substantial progress, ongoing research and more extensive studies are imperative to continually refine acne scar treatment outcomes, ultimately enhancing the well‐being of those affected by this condition.

## 1 Introduction

Acne vulgaris is a persistent inflammatory skin condition that may lead to enduring post‐inflammatory scarring [1]. A recent 2023 study revealed that 47% of participants diagnosed with acne experienced the consequences of acne scars [2].

Severe acne lesions can result in permanent scars, causing psychological distress [3]. The extent and severity of scarring in acne are greater when treatment is delayed [4]. Acne scarring can be categorized into two main types. The first involves increased tissue formation, including hypertrophic and keloid scars. The second, more common type involves tissue loss and includes ice pick, rolling, and boxcar‐type scars [5].

Treating acne scars is a complex process due to numerous variables that must be considered. Different scar morphologies are among the primary factors influencing the treatment approach [6]. The degree of scarring is crucial in determining the appropriate treatment approach, with severe scarring requiring different strategies than mild scarring [7].

The treatment of acne scars remains a controversial topic, with an increasing demand from patients for a less invasive approach with consistent efficacy and fewer side effects [8]. Several studies have demonstrated that treatments, including micro needling and laser procedures, have shown beneficial results in patients with acne scars [9, 10]. Despite recent advances in treatment technologies, comprehensive studies have not fully caught up with the current knowledge and practices in many centers. In this study, our focus was on advancements in acne scar treatment and the exploration of novel therapies in research.

## 2 Methods

### 2.1 Search Strategy

The search was performed within the following databases: PubMed, Scopus, and ScienceDirect, and articles published between 2013 and 2023 were included. To mitigate the occurrence of missing data, a search was conducted on Google Scholar using the keywords “Acne Vulgaris,” “Acne Keloid,” “Scar,” and “Therapy.”

The inclusion criteria comprised studies published in English with full text available, involving participants aged 10–65 years diagnosed with acne scars at any stage of the disease. Included interventions were those aimed at treating acne scars. However, studies with interventions specifically targeting homeless patients, participants with physical or mental handicaps, and individuals involved in the forensic process were excluded. Gray literature that did not manifest as articles, such as posters, organizational projects, class presentations, health messages, and similar literature, did not meet the eligibility criteria.

### 2.2 Data Extraction

To identify eligible studies, two independent experts assessed titles and abstracts from databases and additional sources. Subsequently, the same two experts reviewed the retrieved full texts. Any disagreements were resolved through consultation with a third expert. In cases where data were unavailable, requests were made to the study authors. Two experts were tasked with the data extraction process for the retrieved studies. The following items were extracted:

1. General information (Author, publication year, reference, study type, sample size)
2. Participants
3. Measurement tool
4. Intervention period
5. Outcome
6. Main finding or key points
7. A summary of methods and results is presented in Table 1.

**TABLE 1:** Summary of methods and results.

Method	Details
Databases searched	PubMed, Scopus, ScienceDirect, Google Scholar
Keywords used	“Acne Vulgaris,” “Acne Keloid,” “Scar,” “Therapy”
Publication date range	2013–2023
Inclusion criteria	Studies published in English, full text available, participants aged 10–65 years diagnosed with acne scars, interventions aimed at treating acne scars
Exclusion criteria	Studies targeting homeless patients, participants with physical or mental handicaps, individuals involved in the forensic process, gray literature not manifesting as articles (e.g., posters, organizational projects, class presentations, health messages)
Data extraction	Conducted by two independent experts, with disagreements resolved by a third expert. Extracted items included: 1. General information (Author, publication year, reference, study type, sample size) 2. Participants 3. Measurement tool 4. Intervention period 5. Outcome 6. Main finding or key points
Initial search results	PubMed: 259 records, Scopus: 226 records, ScienceDirect: 215 records, Google Scholar: 189 records
Post‐duplicate removal	625 manuscripts remained
Title review results	321 eligible articles
Abstract screening	74 manuscripts retrieved
Final selection	26 studies met inclusion criteria and were reviewed, involving 702 participants
Study type	
Almost all were interventional studies	

## 3 Results

The initial search generated 259 records from PubMed, 226 from Scopus, and 215 from the ScienceDirect databases. Additionally, 189 articles were identified through Google Scholar. After eliminating duplicates, 625 manuscripts remained. Through title review, the pool of eligible articles was narrowed down to 321. Subsequent abstract screening resulted in retrieving 74 manuscripts. The primary objective of the study was to select research focusing on various treatment methods for acne scarring, leading to a substantial reduction in eligible articles. Ultimately, 26 studies met the inclusion criteria and underwent review. Table 1 illustrates the study selection process. Almost all studies were interventional, encompassing a total of 702 participants in this comprehensive literature review. Further details and key findings are presented in Tables 2 and 3.

**TABLE 2:** Overview of included studies.

Author, year, ref.	Sample size	Study type	Participants	Measurement tool	Intervention period	Control	Outcome	Key points/main findings
Bhat et al. (2023) [11]	(*n* = 104)	Hospital‐based observational study	Individuals aged ≥ 18 years, experiencing atrophic acne scars on their faces for more than 6 months	Goodman and Baron's qualitative scar scale	6 months	N/A	Resurfacing in atrophic facial acne scars	The application of fractional ablative CO_2_ laser resurfacing has shown remarkable results in effectively treating atrophic acne scars
Bazargan et al. (2023) [12]	(*n* = 17)	Clinical trial	Patients with persistent post‐acne erythema	Visioface device	3 months	Left side of each patient's face	Treatment of post‐acne erythema	Tranexamic acid injection administered as mesotherapy exhibited statistically significant improvements in lesion count, area, and area percentage on the right side of the face (case group), while no significant changes were observed on the left side (control group).
Lotfi et al. (2023) [13]	(*n* = 30)	A pilot investigative study	Patients suffering from acne scars	Goodman and Baron scores (GBA), Patient's Global Assessment (PGA), Investigator's Global Assessment (IGA)	7 months	N/A	treating acne scars	A single session of endo‐radiofrequency subcision proves to be a safe and effective treatment for acne scars, yielding high patient satisfaction rates among those who undergo the procedure.
Shoaib et al. (2023) [14]	(*n* = 50)	Pilot clinical trial	Patients suffered from mild to severe rolling acne scars	Demographic information, information on any medical conditions	17 months	N/A	Treatment of post acne rolling scars	In over 80% of patients, platelet‐rich plasma (PRP) and radiofrequency (RF) microneedling demonstrated effectiveness as treatments for atrophic acne scars, while causing only a few adverse effects.
Mamizadeh et al. (2023) [15]	(*n* = 30)	RCT	Patients with bilateral grade 3–4 acne scars	Researcher‐made questionnaire, Goodman and Baron's scar grading scale	1 month	N/A	Improve acne scars	After 1 month of treatment, microneedling combined with subcision shows promising and satisfactory results for patients with grade 3–4 acne scars, outperforming the combination of CO_2_ fractional laser and subcision.
Eubanks et al. (2022) [16]	(*n* = 23)	A prospective study	Subjects with moderate to severe acne scarring	Visual Analog Scale (VAS) Subject, Satisfaction Questionnaires, Global Aesthetic Improvement Scale (GAIS)	1 month	N/A	Acne Scarring Improvement, Subject Satisfaction	Fractional radiofrequency (FRF) showed a statistically significant improvement in acne scarring, as assessed by independent blinded evaluators using the GAIS scale.
Abdel et al. (2021) [17]	(*n* = 33)	A split‐face clinical trial	Patients with atrophic acne scars	Subjective evaluation by clinical assessment, Objective evaluation using skin biopsies for histological and quantitative molecular analysis	3 months	N/A	Quality of acne scars	The application of topical stem cell‐conditioned medium (SC‐CM) has the potential to enhance the effectiveness of fractional carbon dioxide laser (FCL) treatment for atrophic acne scars.
An et al. (2020), [18]	(*n* = 45)	RCT	Patients with acne scars on both the cheeks	Visual Assessment and Patient Satisfaction, Objective Scar Assessment		N/A	Quality of acne scars	The combination therapy involving Poly‐lactic acid with MFRF resulted in significantly improved acne scar assessment scores and higher patient satisfaction compared to the monotherapy using MFRF with normal saline (*p* = 0.036 and *p* = 0.009, respectively).
Al‐Hamamy et al. (2020) [19]	(*n* = 30)	RCT	Patients with acne scars	Score ranging from 0 to 20	3 months	N/A	Treatment of mild and moderate acne scars	The combination of a superficial peel using trichloroacetic acid, followed by manual dermasanding in separate sessions, demonstrated effectiveness in treating mild and moderate acne scars.
Joseph ET AL. (2019) [20]	(*n* = 42)	Clinical trail	Patients with acne scars	Acne Scar Assessment Scale, Global Aesthetic Improvement Scale, Physician GAIS	7 months	N/A	Full‐face acne scar treatment	Four months after the initial treatment, 92% of the subjects exhibited a response, showing an improvement of at least one point on the Acne Scar Assessment Scale. At the 7‐month mark, this percentage further increased to 95%.
Al Taweel et al. (2019) [21]	(*n* = 40)	Clinical trial	Patients with acne scars	Visual examination by the physicians or researchers	3 months	N/A	Improvement in skin texture and tone	Fractional CO_2_ laser demonstrated a significantly superior level of improvement compared to carboxytherapy in the treatment of acne scars. Carboxytherapy showed promising results for treating acne scars with fewer complications.
Chen et al. (2019) [22]	(*n* = 78)	Clinical trial	Patients with acne scars	Self‐evaluation by the patients themselves/evaluation by clinicians or researcher	2 years	Dermabrasion alone	Treatment on the therapy of acne scars	The combination of ReCell autologous cell regeneration techniques with dermabrasion was evaluated for the treatment of acne scars. The group that received BoNTA (Botulinum toxin A) showed more significant and statistically noticeable improvements in comparison to the control group, as observed through SBSES (Scar Cosmesis Scale) and VAS (Visual Analog Scale).
Kim et al. (2019) [23]	(*n* = 45)	RCT	Patients with forehead laceration	Observer scar assessment scale, Stony Brook scar evaluation scales (SBSESs), and visual analog scale (VAS)	6 months	Receiving saline	Scar formation, collagen deposition	Improvement in the functional, emotional, and cosmetic aspects of scar formation in the groups receiving BoNTA treatment was demonstrated. After surgery, burns, or trauma, the use of BoNTA may be increased to prevent hypertrophic scarring.
Tawfik et al. (2019) [24]	(*n* = 24)	RCT	Patients with severe HTS lesions	Vancouver scar scale (VSS)	8 months	N/A	Treating severe hypertrophic scars	Topical 5‐FU is a conventional therapy for hypertrophic scars (HTS).
Kareem et al.(2019) [25]	(*n* = 20)	Comparative split face study	Patients with atrophic acne scars	Standardized photographs	3 months	N/A	Treatment of atrophic acne scars.	The combination of subcision using carboxytherapy with fractional carbon dioxide laser resurfacing resulted in a more significant improvement in atrophic acne scars compared to using fractional laser resurfacing alone.
El‐Domyati et al. (2018) [26]	(*n* = 24)	Split‐face clinical trial	Volunteers with postacne atrophic scars	Five‐point scale Photography and punch biopsies.	3 months	Dermaroller alone was used	Quality of acne scars	Despite the fact that the majority of volunteers showed a substantial improvement following treatment, the clinical, histometrical, and histochemical evaluation revealed that the combination of dermarolling and trichloroacetic acid 15% peeling was superior to dermarolling plus PRP or dermarolling alone for treating post‐acne atrophic scars.
Nor et al. (2017) [27]	(*n* = 17)	RCT	Patients with keloid	Patient and Observer Scar Assessment Scale	3 months	N/A	Treatment of keloid	Clobetasol propionate 0.05% cream, when applied under occlusion with silicone dressing, is equally effective in treating the condition and has fewer adverse effects compared to intra‐lesional (IL) triamcinolone.
Kar et al. (2017) [28]	(*n* = 30)	A Split‐face comparison trial	Patients with moderate‐to‐severe acne scars	Goodman and Baron's quantitative scar scale, Observer visual scar assessment scale, Patient visual scar assessment scale, Visual disease severity scale	17 months	Receiving FCL alone	Quality of acne scars	Both fractional carbon dioxide laser (FCL) alone and the combination of FCL with platelet‐rich plasma (PRP) were effective in managing acne scars and led to a significant improvement in scar quality.
Kwon et al. (2017) [29]	(*n* = 28)	RCT	Patients with atrophic acne scars	ECCA, Investigator's Global Assessment	16‐week	Nonablative 1550‐nm erbium‐glass laser	Quality of acne scars	The combination therapy of nonablative 1550‐nm erbium‐glass laser and microneedling demonstrated greater improvement compared to nonablative 1550‐nm erbium‐glass laser used alone as a monotherapy.
Meymandi et al. (2016) [30]	(*n* = 166)	Clinical trial	Patients with atrophic acne scars	Vancouver scar scale criteria	3 weeks	Cryotherapy method with corticosteroid intralesional injection	Treatment of scars, improvement level, complications, and patient satisfaction	The study results indicated that the recovery rate was higher in the Cryotherapy group than in the IPL (Intense Pulsed Light) group, but this difference did not reach statistical significance (*p* > 0.05)
You et al. (2016) [10]	(*n* = 58)	A retrospective photographic analysis	Patients who underwent laser treatment for facial atrophic acne scars	Scale graded from 0 to 10	20 years	N/A	Facial atrophic acne scars	The CO_2_ laser, Er:YAG laser, and AFL (ablative fractional laser) all demonstrated effectiveness in improving acne scars. However, the CO_2_ laser resulted in longer downtime compared to the other lasers.
Faghihi et al. (2016) [31]	(*n* = 16)	A split‐face randomized clinical trial	Patients who underwent split‐face therapy	Using a digital camera, using a quartile grading system for satisfaction, side adverse events by a blinded dermatologist	4 months	N/A	Patient satisfaction	The overall clinical improvement of acne scars was observed to be higher on the side treated with PRP‐fractional carbon dioxide laser. However, the difference was not statistically significant at both the 1‐month follow‐up after the first treatment session (*p* = 0.15) and the 4‐month follow‐up after the second session (*p* = 0.23).
Cachafeiro et al. (2016) [32]	(*n* = 46)	RCT	Patients with atrophic facial acne scars	Quantitative Global Grading System for Postacne Scarring	6 months	N/A	Treatment of atrophic acne scars	The study provides evidence that both nonablative fractional laser 1340 nm and microneedling are effective treatments for atrophic acne scars.
Faghihi et al. (2015) [31]	(*n* = 46)	RCT	Subjects with Fitzpatrick skin types III to IV and moderate to severe atrophic acne scars on both cheeks	Two dermatologists	4 months	N/A	Treatment of atrophic acne scars	The combination of fractional laser skin resurfacing and punch elevation proves to be a safe and effective approach for addressing acne scarring.
Klinger et al. (2014) [33]	(*n* = 40)	A pilot study	Patients with moderate to severe atrophic acne scars	Questionnaire Cardiff acne disability index (CADI)	6 months	N/A	Treating atrophic acne scars	Autologous bone marrow stem cells appear to be a secure and efficient treatment option for managing all types of atrophic facial acne scars.
Bjørn et al. (2014) [34]	(*n* = 13)	RCT	Patients with symmetrical atrophic acne scars on right and left sides of the mid‐face and lower‐face	A 10‐point scale for evaluating acne scars	One group received fractional CO_2_ laser treatments at 1‐month intervals, and the other group received treatments at 3‐month intervals.	N/A	Scar atrophy, patient satisfaction, and adverse effects.	The reduction in scar atrophy was evident at both 1 month and 6 months after the surgery, irrespective of whether the treatment interval was 1 month or 3 months

Abbreviations: N/A, not applicable; NR, not reported; RCT, randomized controlled trial.

**TABLE 3:** Correlation between acne treatment modalities, type of acne, and ethnicity/skin type considerations.

Type of acne	Treatment modalities	Effectiveness	Ethnicity/skin type
Comedonal acne (blackheads and whiteheads)	– Topical retinoids (tretinoin, adapalene) – Benzoyl peroxide Salicylic acid	Promotes cell turnover, prevents clogged pores	Skin of color (Fitzpatrick IV‐VI): – Use gentler retinoids (e.g., adapalene) to minimize irritation and hyperpigmentation. – Avoid aggressive treatments. Fair skin (Fitzpatrick I‐III): – Standard retinoids – Lower concentration benzoyl peroxide to minimize irritation. Asian skin: – Gentle cleansers and moisturizers to support skin barrier function. Hispanic skin: – Combination therapy with retinoids and benzoyl peroxide. Emphasize sunscreen use to prevent hyperpigmentation.
Inflammatory acne (papules and pustules)	– Topical antibiotics (clindamycin, erythromycin) – Benzoyl peroxide – Topical and oral retinoids Oral antibiotics (doxycycline, minocycline)	Reduces bacteria and inflammation	Skin of color (Fitzpatrick IV‐VI): – Azelaic acid to reduce inflammation and pigmentation. – Avoid high concentration benzoyl peroxide. Fair skin (Fitzpatrick I‐III): – Topical antibiotics – Milder benzoyl peroxide. Asian skin: – Use azelaic acid for anti‐inflammatory and depigmenting properties. Hispanic skin: – Combine retinoids with benzoyl peroxide for balanced treatment. Emphasize sunscreen use.
Nodulocystic acne (nodules and cysts)	– Oral isotretinoin – Oral antibiotics Intralesional corticosteroid injections	Isotretinoin is particularly effective for severe cases, providing long‐term remission	Skin of color (Fitzpatrick IV‐VI): – Close monitoring for side effects. – Use azelaic acid for pigmentation control. Fair skin (Fitzpatrick I‐III): – Standard isotretinoin protocols. Asian skin: – Cautious use of isotretinoin, monitoring for sensitivity. Hispanic skin: Emphasize combination treatments and monitoring.
Hormonal acne	– Oral contraceptives – Anti‐androgens (spironolactone) – Retinoids Benzoyl peroxide	Effective for acne related to hormonal fluctuations	Skin of color (Fitzpatrick IV‐VI): – Hormonal treatments combined with skin‐lightening agents to prevent PIH. Fair skin (Fitzpatrick I‐III): – Standard hormonal treatments. Asian Skin: – Hormonal treatments with supportive skincare. Hispanic Skin: – Emphasize hormonal balance and sunscreen use.

## 4 Discussion

The treatment of acne scars involves the use of a variety of methods, each chosen depending on the type of scar to be treated.

### 4.1 Laser and Light Therapies

The integration of lasers, light‐based technologies, and energy‐based approaches has become essential in the field of acne and acne scar treatments. This encompasses a diverse range of lasers, including those using infrared wavelengths and pulsed dye lasers (PDLs), as well as light devices such as blue light, red light, and broadband light [10]. The management of acne scars utilizes different types of lasers, which can be classified as either ablative or non‐ablative. Ablative lasers, including the carbon dioxide laser and Erbium YAG laser, work by focusing on water absorption to vaporize and eliminate damaged scar tissue. On the other hand, non‐ablative lasers, such as NdYAG and Diode lasers, stimulate dermal fibroblasts to encourage the generation of new collagen. An example of a non‐ablative laser system used in this context is the 675‐nm RedTouch laser from Deka Me.La, Italy [14, 24, 34]. In a study, researchers found that Fractional CO_2_ (FX CO_2_) laser resurfacing is effective in enhancing atrophic acne scars. Significant improvements were observed in acne scar atrophy both 1 month and 6 months after the procedure, regardless of the treatment interval. The interval between treatments did not impact the degree of improvement in scar atrophy, patient satisfaction, or the occurrence of postoperative adverse effects [34].

Recent clinical perspective emphasizes integrating lasers, light‐based, and energy‐based technologies in treating acne and acne scars. Both ablative (e.g., CO_2_, Erbium YAG) and non‐ablative lasers (e.g., NdYAG, Diode) effectively enhance scar appearance, with FX CO_2_ laser resurfacing showing significant, consistent improvements in atrophic acne scars. A great piece of literature approves the efficacy and safety of laser treatments for acne scars; however, side effects including risk of skin infection, inflammation, hypersensitivity reactions, and keloids have been associated with this treatment method [24].

### 4.2 Microneedling

Microneedling is a technique that utilizes fine needles to create numerous tiny holes in the skin. These minuscule punctures, made by the micro‐pen machine, stimulate the skin's natural regenerative process, leading to the production of collagen and elastin [9]. Consequently, this method effectively improves wrinkles, fine lines, pimples, wounds, and pores. However, a significant issue with microneedling is that clinical outcomes often depend on subjective assessments by physicians and/or volunteers. Furthermore, the histopathological responses following microneedling treatment are relatively limited [19]. Microneedling effectively treats wrinkles, fine lines, and scars by stimulating collagen production, though outcomes often rely on subjective assessments, which typically involve physicians or volunteers evaluating the improvement in skin appearance, such as reduction in wrinkles, fine lines, and scars, based on personal judgment rather than objective measurements.

### 4.3 Chemical Peels

A commonly employed method for treating acne and acne scars is chemical peeling. This procedure involves the controlled removal of superficial skin lesions by exfoliating and eliminating a portion or the entire epidermis, with or without the dermis [26]. The process stimulates the regeneration of new epidermal and dermal tissues. Various peeling agents are available, with the most popular ones being salicylic acid, glycolic acid, pyruvic acid, lactic acid, mandelic acid, Jessner solution, trichloroacetic acid, and phenol. Each of these agents has unique characteristics and indications for treating various skin conditions and types of scars [9, 35].

A specific study demonstrated the effectiveness of a trichloroacetic acid superficial peel followed by manual dermasanding in separate sessions for treating mild and moderate acne scars. The improvement became more significant with repeated dermasanding sessions [26]. The patient's skin type, acne activity, and the type of acne scarring all influence the choice of the best chemical peel. Combination peels are frequently chosen because they help reduce the possibility of negative effects [36].

Chemical peels can be used in conjunction with other procedures to treat acne scars and produce better clinical results [37]. They work by removing the topmost layer of the skin, and by promoting skin renewal, they can help improve the appearance of mild to severe acne scars. However, it is worth noting that chemical peels may not significantly improve cases of more extensive scarring that extends deeper into the skin [36].

The effectiveness of these methods in promoting skin renewal and improving scar appearance is of increasing importance in recent clinical practice. Various peeling agents, such as salicylic acid and trichloroacetic acid, are tailored to specific skin types and scar conditions.

Generally, glycolic acid provides effective superficial exfoliation and texture improvement but may not suffice for deeper scars and can irritate sensitive skin. Salicylic acid penetrates pores to combat active acne, although its drying effect can be a downside. Lactic acid is gentler and hydrating, making it suitable for sensitive skin, but may also deliver slower results. Medium‐depth peels, like trichloroacetic acid and Jessner's solution, can significantly enhance skin texture but come with downtime and potential risks of irritation and hyperpigmentation. For deep scars, phenol peels offer profound and long‐lasting effects, though they require extensive recovery time and can lead to complications. Retinoids, while effective for both acne and scarring, demand consistent use and sun protection, while enzyme peels provide gentle exfoliation with minimal downtime but may be less effective on deeper scars. Combining peels with other treatments enhances results, though deeper scars may need additional approaches [9, 26, 35, 36, 37].

### 4.4 Dermal Fillers

Dermal fillers have become essential in both medical and cosmetic dermatology. These fillers are categorized as temporary, semipermanent, and permanent [20]. The first two classes are biodegradable and have varying half‐lives, while permanent fillers are non‐biodegradable. When using fillers, they prompt a distinctive response in the extracellular matrix (ECM), enabling the identification of different filler types through histopathological examination [38]. The use of dermal fillers in the treatment of atrophic acne scars is supported by data from clinical research. These fillers include silicone, collagen, hyaluronic acid, polymethylmethacrylate (PMMA)‐collagen gel, polyacrylamide, poly‐L‐lactic acid (PLL), and calcium hydroxylapatite (CaHA). The tolerability and safety of dermal filler implantation are influenced by the filler's properties, the doctor's skill and knowledge, and the patient's unique traits. Dermal filler injections to improve acne scars entail a soft‐tissue augmentation procedure [39]. Collagen synthesis is boosted by hyaluronic acid fillers (HAFs). However, semipermanent or biostimulatory fillers such as PLL and CaHA, as well as permanent fillers, are used to promote collagen formation more forcefully [20].

These agents play a crucial role in soft‐tissue augmentation and collagen synthesis. Temporary, semipermanent, and permanent fillers like hyaluronic acid, PLL, and CaHA are used based on their properties and patient needs. Fillers improve atrophic acne scars, with safety and effectiveness depending on filler type, physician expertise, and patient characteristics.

### 4.5 Platelet‐Rich Plasma (PRP) Therapy

Limited research indicates that PRP can be used as an adjuvant therapy for atrophic acne. Autologous PRP is obtained from the patient's blood and contains a concentrated number of platelets [19]. PRP acts as a supplementary therapy for treating atrophic acne scars alongside main therapies, including microneedling, fractional carbon dioxide laser (FCL), and subcision [40]. One study showed that autologous PRP can be introduced as a cost‐effective and well‐tolerated office procedure for treating acne scars without significant side effects [41].

The impact of PRP on the treatment of acne scars may be attributed to its ability to expedite the generation of hyaluronic acid [42]. Hyaluronic acid draws water into its matrix, resulting in swelling, increased volume, improved skin turgor, and enhanced tissue lubrication [40]. Additionally, native hyaluronic acid has been shown to promote cell proliferation, stimulate ECM synthesis, and regulate collagen fiber diameter, leading to improvement in atrophic scars [43].

PRP is a promising adjuvant treatment, which enhances main therapies like microneedling and lasers by promoting hyaluronic acid generation, which improves skin volume and texture. PRP is cost‐effective, well‐tolerated, and boosts collagen synthesis, aiding in scar improvement. There is an ongoing interest in adopting this effective method for the treatment of various dermatological disorders by dermatologists and patients.

### 4.6 Subcision

S. Orentreich and N. Orentreich created subcutaneous incision‐less surgery, sometimes referred to as subcision, as a less invasive method to repair acne scars in 1995 [44]. The scar's fibrotic strands must be broken, and the epidermis must be separated from the underlying connective tissue. This procedure causes the growth of new connective tissue, elevation of the acne scars' recessed surface and enhances their appearance [42]. Vempati et al.'s retrospective analysis revealed that subcision was a successful method of treating atrophic acne scars. The research revealed four main subcision tool types: needles, cannulas, wires, and blunt‐blade instruments. These tools are widely used to treat atrophic acne scars. The depth of the scars, a person's preferences, and the particular mix of therapies being utilized are among the elements that influence the instrument selection [25].

While needle‐based subcision using conventional hypodermic or Nokor needles has been effective, its utilization is limited due to various issues. These include the need for multiple insertion sites, discomfort for patients, potential damage to neurovascular structures, needlestick injuries, and bleeding [19]. Additionally, one should take into account any post‐operative adverse effects such as bruising, hematoma, scarring, nodule formation, and post‐inflammatory hyper‐ or hypopigmentation [16]. Various tools—needles, cannulas, wires, and blunt‐blade instruments—are used based on scar depth and patient needs. Despite its success, needle‐based subcision poses risks such as discomfort, neurovascular damage, and postoperative effects like bruising and pigmentation changes.

### 4.7 Radiofrequency Microneedling (RFM)

RFM represents a significant advance in conventional radiofrequency (RF) devices when it comes to skin tightening, offering enhanced safety and efficacy. This technique enables efficient energy delivery into the dermis while causing minimal disruption to the epidermis, especially when using insulated needle devices [18].

It involves a controlled skin injury that triggers the development of rejuvenated and healthy skin and benefits common skin problems, including acne scars and wrinkles. The use of devices that monitor tissue characteristics, including temperature and impedance, further contributes to optimizing patient outcomes and ensuring safety during the procedure [13].

Given its benefits, RFM should be considered the new standard for treating acne scarring, particularly in individuals with darker skin types. Additionally, it is an effective minimally invasive solution for neck rejuvenation and treatment of skin laxity in this area [13, 45]. A study indicated that treating moderate and severe acne scars with microneedling and fractional RF is effective [30].

RFM is considerably advanced over conventional RF devices in skin tightening, particularly for acne scars and wrinkles. RFM ensures efficient energy delivery with minimal epidermal disruption, especially with insulated needles. This technique promotes rejuvenated skin development and optimizes patient outcomes through tissue monitoring. RFM is especially recommended for treating acne scars in darker skin types and for neck rejuvenation, offering a minimally invasive solution for skin laxity. Studies confirm its effectiveness for moderate to severe acne scars.

### 4.8 Cryotherapy

Cryotherapy is a treatment method utilizing liquid nitrogen to freeze the affected area, causing the breakdown and destruction of scar tissue. It is a commonly used physical approach to treat acne and has been used for many decades. Research has been conducted on the use of contact cryotherapy for treating hypertrophic acne scars. Though the precise mechanism for scar reduction is still unknown, one idea contends that the physical harm brought on by freezing and thrombosis causes collagen to remodel, which improves the scars [5, 46]. The main disadvantage of cryotherapy is the formation of permanent light spots on the treated skin. Additionally, it is essential to recognize that cryotherapy may not be effective for all types of acne scars, as the most suitable treatment option depends on the specific type of acne scar [47].

This method is associated with a long‐standing use and ongoing research, particularly in treating hypertrophic scars. While its mechanism for scar reduction is not fully understood, it likely involves collagen remodeling due to freezing‐induced physical damage. However, the formation of permanent light spots is a notable disadvantage. Patient acceptance may vary due to the risk of side effects, while dermatologists may consider cryotherapy as one of several options depending on scar type and patient preference.

### 4.9 Fluorouracil (5‐FU)

The use of 5‐FU for scar treatment was initially proposed by Fitzpatrick in 1999. Studies have confirmed that 5‐FU tattooing is more effective than intralesional corticosteroids alone. However, the most effective approach for scar treatment appears to be the combination of 5‐FU injections with intralesional corticosteroids and PDL treatments [14, 22].

### 4.10 Dermabrasion

Dermabrasion is a procedure involving manual sanding of the epidermis and upper dermis to remove them. It can be done using hydrogen peroxide and sandpaper to control bleeding, or with a rotating motorized handpiece equipped with various tools including serrated wheels, diamond‐embedded fraises, and wire brushes. The removal of superficial skin layers during this process promotes a smoother wound‐healing process and stimulates the formation of new collagen [48].

Dermabrasion is a skin‐resurfacing technique aimed at enhancing the appearance of acne scars and other skin imperfections. This procedure employs a rapidly rotating device to eliminate the outer layer of the skin, facilitating the growth of smoother and rejuvenated skin [27]. Dermabrasion can be performed as a standalone procedure or in combination with other cosmetic treatments. However, there is a rare risk of infection or scarring associated with dermabrasion. In cases where scarring occurs, steroid medications can be utilized to help minimize their appearance as a result of the dermabrasion procedure [48].

Dermabrasion can be performed alone or in combination with other cosmetic procedures. However, there are risks of infection and scarring, albeit rare. Patient acceptance may vary due to these risks, while dermatologists consider dermabrasion a viable option for scar treatment, especially when combined with other modalities for enhanced results.

### 4.11 Silicone Dressings

With their efficiency and low risk of side effects (AEs), silicone dressings offer a practical and efficient solution for treating hypertrophic acne scarring. These dressings are thin silicone gel or membrane sheets placed topically on acne scars [49]. Their therapeutic impact is attributed to the synergistic effect of pressure and moisture, inhibiting collagen formation by fibroblasts. The only negative effects linked to silicone dressings are localized skin maceration and pruritus (itching). Though silicone dressings are beneficial for various types of scars beyond hypertrophic acne scars, such as post‐surgical scars and burn scars, as they help flatten and soften scar tissue while promoting optimal healing in a hydrated environment. Additionally, they can improve scar color and texture, reduce itchiness, and are suitable for sensitive skin, making them a versatile option in scar management. It is important to keep in mind that patient compliance may be a challenge, particularly when dressings must be applied to highly visible locations including the face. Some patients may choose to apply the dressings at night, but doing so may lessen their effectiveness [5, 50]. Using silicone dressings for hypertrophic acne scarring highlights their efficacy and low risk of side effects, primarily localized skin maceration and itching. Dermatologists generally accept silicone dressings as a practical and efficient treatment option, especially for less visible scars. However, patient compliance, particularly when applied to highly visible areas like the face, can pose challenges. Some patients may opt to apply dressings at night, potentially reducing their effectiveness.

### 4.12 Mesotherapy

Mesotherapy is a cosmetic procedure involving the injection of a blend of vitamins, minerals, and nutrients into the skin to rejuvenate and repair it, making it highly effective in treating acne scars and various skin issues. This treatment provides targeted wound repair and skin regeneration, making it suitable for all types of scars [51]. Often combined with microneedling, creating tiny punctures in the skin to stimulate the body's natural healing process and enhance collagen and elastin production, mesotherapy further aids in reducing the appearance of acne scars [52]. However, it is essential to consider that mesotherapy is not recommended for individuals with specific conditions, including active acne, eczema, psoriasis, or rosacea. Additionally, those prone to easy bruising or having blood disorders, HIV, herpes, chronic illnesses, or recent surgery within the past 6 months may not be suitable candidates for this treatment [53]. However, patient acceptance may vary, as mesotherapy is not recommended for individuals with specific conditions like active acne or certain medical histories, which may limit candidacy.

### 4.13 Carboxytherapy

Despite not being a new technique, carboxytherapy, which involves the controlled administration of CO_2_ in intradermal or subcutaneous doses, is increasingly gaining recognition among esthetic medicine doctors, dermatologists, and cosmetologists worldwide [21]. Carboxytherapy shows promise as a valuable tool in the treatment of atrophic acne scars [54]. In a study evaluating acne scar treatment, carboxytherapy and microneedling were compared; 32 patients received six sessions of each treatment on opposite sides of the face, resulting in a significant reduction of all acne scar types (icepicks, boxcar, and rolling) for both procedures [55].

Carboxytherapy is considered a safe method for managing acne scars. The associated side effects are minor, with the most common complication being small bruises at the sites of needle insertions. However, these bruises usually disappear within a few days without leaving any lasting marks [56]. This is highly accepted by patients, dermatologists, and esthetic medicine doctors.

### 4.14 Intralesional Therapy

Intralesional corticosteroid injections are a widely used and effective treatment for hypertrophic acne scarring. Their mechanism of action involves reducing fibroblast proliferation and promoting collagen degradation [57]. However, there are potential side effects associated with corticosteroid injections, including the development of hypopigmentation, dermal atrophy, and telangiectasia [5]. In cases where corticosteroid treatment proves ineffective, other intralesional options such as 5‐fluorouracil, bleomycin, and verapamil have been used with positive outcomes. These agents work by inhibiting the proliferation of dermal fibroblasts [58].

### 4.15 Punch Techniques

Punch methods are effective for treating cases of severe atrophic acne scarring, especially when other methods have not been very successful. The procedure involves a punch excision, where a circular incision is made approximately equal to the size of the scar. Depending on the case, the scar tissue may be removed and then sutured, replaced with a graft, or elevated to match the surrounding skin level using sutures or adhesive skin closure material [59, 60]. Punch techniques have been demonstrated to be successful, notably for treating ice‐pick scars, even if the success rates of this procedure are mostly based on case studies [5]. The dangers of this strategy, meanwhile, include graft failure, graft depression, and the development of sinus tracts. But when done correctly, punching techniques present a viable approach to treating more severe atrophic acne scars [61].

### 4.16 Soft‐Tissue Augmentation

Soft‐tissue augmentation, which involves injecting collagen fillers including hyaluronic acid, CaHA, PLL, silicone, and autologous fat, is a successful treatment for superficial atrophic acne scarring. By stretching dermal fibroblasts, these fillers restore lost tissue volume and promote collagen formation. For the best cosmetic outcomes, multiple treatments might be necessary, although they have proven to be quite effective in healing atrophic acne scars. Rolling scars respond particularly well to hyaluronic acid [5, 62]. It should be emphasized that these procedures only provide transitory results, necessitating additional visits to maintain the desired cosmetic results. Recently approved treatments include autologous fibroblast transfer and PMMA microspheres suspended in bovine collagen for people seeking more long‐lasting remedies [60]. This is a successful treatment for atrophic acne scars, with good acceptance among patients and dermatologists.

### 4.17 Stem Cell Therapy

For skin and scar regeneration, using adult stem cells (SCs) is becoming more and more practical. Bone marrow (BM), adipose tissue, the umbilical cord, and skin tissue are just a few of the tissues from which SCs can be obtained [33]. The potential for BM‐derived SCs (BMSCs) to aid in tissue repair or regeneration in a variety of tissues, including the heart, blood vessels, broken bones, tendons, cartilage, and skin, has been demonstrated. It is clear that BMSCs are naturally present in healthy skin and that they play a part in host defense, inflammatory responses, and epidermal formation. Researchers are investigating the use of autologous agents as dermal fillers for repairing scars and wrinkles as a result of this increased interest [63]. A novel approach for treating acne scars, SC therapy, has shown encouraging outcomes. A topical SC‐conditioned medium (SC‐CM) may improve the effectiveness of FCL in treating atrophic acne scars, according to one study [64]. Further study is necessary to fully evaluate the efficiency and safety of SC therapy for acne scars, though, as it is still in its early phases of development [65]. Patient acceptance is influenced by the novelty of the approach and the need for more evidence regarding its effectiveness. Dermatologists are likely to monitor developments in SC therapy for scar treatment but may approach it cautiously until more data is available regarding its benefits and risks.

### 4.18 Combinational Method

The effectiveness of combination therapy for healing acne scars has been investigated in numerous studies [50, 66]. For instance, in a meta‐analysis of research, PRP and an ablative FX CO_2_ laser were combined to treat acne scars. Results from exploratory clinical trials showed that when the laser and PRP were combined, as opposed to laser treatment alone, patients' improvement rates were noticeably higher [67]. PDL and FX CO_2_ laser therapy are two more treatments for acne scars that have been the subject of studies [31], fractional carbon dioxide (CO_2_) laser resurfacing with punch elevation [68], fractional erbium‐YAG laser and PRP [11], microneedle fractional RF and topical polylactic acid [13, 69], and autologous cell regeneration techniques with dermabrasion [48]. In general, in most studies that utilized a combination of the mentioned therapeutic methods, the results have shown that the combination of these methods leads to a significantly stronger improvement in treatment outcomes compared to individual treatments.

## 5 Limitations

Despite the efforts to present a perfect literature review, there are still some shortcomings. The studies were included in this study if they were published in English owing to the inclusion criteria. Also, the full text of one article was not available. Additionally, there were various tools for the measurement of outcomes. Although the search strategy was designed precisely and a snowball search of included studies was considered, there may be some missing data. There is a need for conducting more studies to complement the findings of the current study.

## 6 Conclusion

The management of acne scars presents a complex challenge, and diverse therapeutic methods have been developed to address these issues. Laser and light‐based therapies, including FX CO_2_ laser and photodynamic therapy, have shown promising outcomes in treating both acne and acne scarring. Additionally, microneedling, chemical peels, and dermal fillers play significant roles in improving the appearance of acne scars. Other viable options for acne scar reduction include PRP therapy, subcision, RFM, cryotherapy, and fluorouracil treatments, which have all yielded positive results. Combinations of treatments have demonstrated improved effectiveness compared to individual approaches. Soft‐tissue augmentation with fillers including hyaluronic acid, CaHA, and PLL can provide temporary improvement for atrophic acne scarring. The selection of the type of treatment should be based on the type and severity of the condition, as well as the general health of the patients.

## Consent

Written and verbal informed consent has been properly obtained from the patients and their guardians. All content of this research adheres with the ethical guidelines developed by the Committee on Publication Ethics (COPE) during the 2nd World Conference on Research Integrity in Singapore in 2010. All parts of this study meets the Code of Conduct (the Ethics Code) and adheres to the legal requirements of the study country, Iran.

## Conflicts of Interest

The authors declare no conflicts of interest.

## Data Availability

The data that support the findings of this study are available from the corresponding author upon reasonable request.
